# Convergent Evolution of the Seed Shattering Trait

**DOI:** 10.3390/genes10010068

**Published:** 2019-01-19

**Authors:** Valerio Di Vittori, Tania Gioia, Monica Rodriguez, Elisa Bellucci, Elena Bitocchi, Laura Nanni, Giovanna Attene, Domenico Rau, Roberto Papa

**Affiliations:** 1 Dipartimento di Scienze Agrarie, Alimentari ed Ambientali, Università Politecnica delle Marche, via Brecce Bianche, 60131 Ancona, Italy; valeriodivittori@gmail.com (V.D.V.); e.bellucci@univpm.it (E.B.); e.bitocchi@univpm.it (E.B.); l.nanni@univpm.it (L.N.); 2 Scuola di Scienze Agrarie, Forestali, Alimentari e Ambientali, Università degli Studi della Basilicata, viale dell’Ateneo Lucano 10, 85100 Potenza, Italy; tania.gioia@unibas.it; 3 Dipartimento di Agraria, Università degli Studi di Sassari, Via E. De Nicola, 07100 Sassari, Italy; mrodrig@uniss.it (M.R.); attene@uniss.it (G.A.); dmrau@uniss.it (D.R.)

**Keywords:** crop domestication, legumes, common bean, gene expression, pod anatomy, QTL mapping

## Abstract

Loss of seed shattering is a key trait in crop domestication, particularly for grain crops. For wild plants, seed shattering is a crucial mechanism to achieve greater fitness, although in the agricultural context, this mechanism reduces harvesting efficiency, especially under dry conditions. Loss of seed shattering was acquired independently in different monocotyledon and dicotyledon crop species by ‘convergent phenotypic evolution’, leading to similar low dehiscent and indehiscent phenotypes. Here, the main aim is to review the current knowledge about seed shattering in crops, in order to highlight the tissue modifications that underlie the convergent phenotypic evolution of reduced shattering in different types of fruit, from the silique of *Brassicaceae* species, to the pods of legumes and spikes of cereals. Emphasis is given to legumes, with consideration of recent data obtained for the common bean. The current review also discusses to what extent convergent phenotypes arose from parallel changes at the histological and/or molecular levels. For this reason, an overview is included of the main findings relating to the genetic control of seed shattering in the model species *Arabidopsis thaliana* and in other important crops.

## 1. Introduction

Domestication of wild species represents a crucial step for human civilization, in that hunter/gatherer humans became farmers. The impact of domestication on modern crops compared to their wild forms is evident nowadays, as most of the final products (e.g., seeds, fruit, leaves) have undergone dramatic phenotypic changes that are shared between different domesticated species [1]. Indeed, the domestication process involves several morphological and physiological changes that result in genetic, structural, and functional modifications of the domesticated forms. The pattern of changes in crops has been termed the ‘domestication syndrome’ [2]. Among the traits of the domestication syndrome, the loss or reduction of seed shattering is one of the key features in many domesticated species, as it reduces yield losses in the field. However, along with seed dormancy, seed shattering is essential in wild plants to disperse seeds far from the mother plant, in terms of space and time, and thus, to reduce competition between plants of close generations and to increase species fitness.

Seed shattering is an example of convergent phenotypic evolution, and as with other traits of the domestication syndrome, it underwent selection independently in several species, and in different domestication areas and historical periods, leading to the same functional changes. However, seed shattering was lost only partially in several species. For example, snap bean varieties (i.e., stringless types) are completely indehiscent, while all dry beans, which represent most of the common bean production area, have dehiscent pods, which is needed to facilitate threshing [3]. The identification of major and minor genes that control the shattering trait is very important to precisely reconstruct the desired phenotypic architecture of shattering, and this is particularly relevant when wide crosses and different types of domesticated and wild genotypes are used as parents in breeding schemes.

Several studies have been conducted to identify the genes that are directly involved in seed shattering and fruit shedding, which started with the pioneering investigations in *Arabidopsis*, where the intricate regulatory network that underlies silique shattering has been well studied [4,5,6,7,8,9]. Seed shattering was deeply investigated in many crops, both monocotyledons and dicotyledons. Among the main examples, several genes were shown to be involved in the control of seed shattering in rice [10,11,12,13], barley [14], and soybean [15,16]. Genetic control of seed shattering has also been investigated in other legumes, as for example in cowpea [17,18], *Medicago* [19], and common bean, for which quantitative trait loci (QTLs) for pod fiber content and seed shattering have been identified [20,21,22], along with genes that are homologous to those involved in seed shattering in *A. thaliana* [23,24].

One of the most intriguing aspects of studying seed shattering is to determine whether convergent phenotypic evolution was the consequence of parallel adaptive trajectories with mutation and selection at homologous loci, and whether the genetic pathway underlying seed shattering is conserved across species. Moreover, it is worth investigating whether macroscopic convergent phenotypic changes are determined by similar phenotypic modifications at the histological level between closest related species.

This review focuses on the issue of convergent evolution, with an illustration of recent findings on the phenotypic evolution of seed shattering at the histological level. We also aim to provide knowledge about the genetic control of seed shattering in the model species *A. thaliana*, along with the main shattering-related genes that have been identified in important crops.

## 2. Histological Modifications That Underlie Seed Shattering in Crops

**Figure 1:**
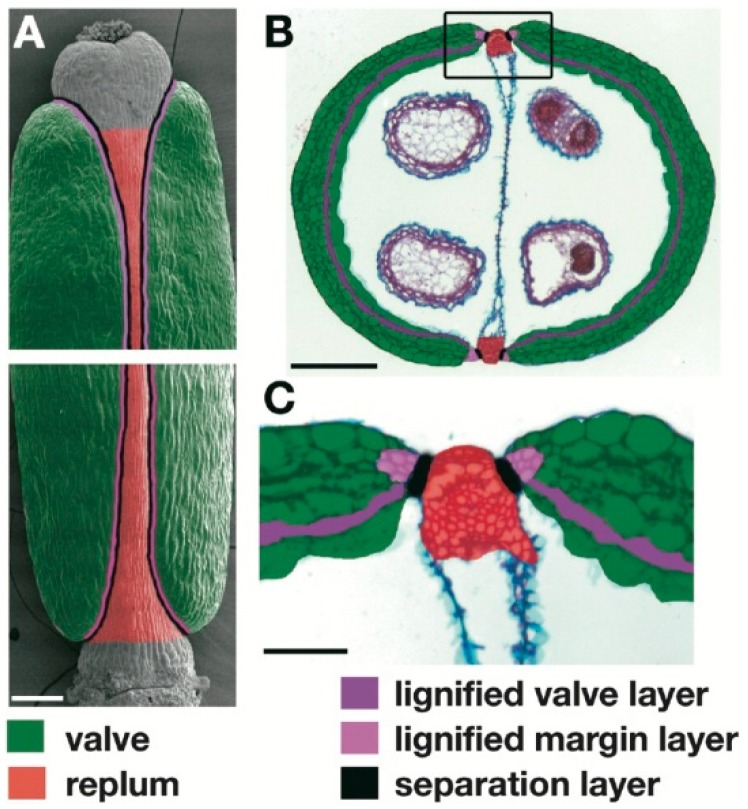
Representative scanning electron micrograph of mature wild-type fruit (stage 17) of *A. thaliana*. (**A**) Apex (top) and base (bottom) of fruit, with regions colored as indicated. (**B**) Transverse section with cell types colored corresponding to (**A**). Box: Valve margin region shown in (**C**). (**C**) Close up of valve margin region. Scale bars: 200 µm, (**A**,**B**); 50 µm; (**C**) (reproduced with permission from Liljegren et al. [8]).

Histological fruit modifications related to seed shattering have been investigated in detail in the *Brassicaceae* family. In the model species *A. thaliana*, the mature silique is formed by three different tissues: the valves, the replum, and the valve margins, which are located between each valve and the replum (Figure 1). The valve margins correspond to the dehiscence zone, and they comprise two further tissues: the lignification layer and the separation layer. The lignification layer at the valve margin and an internal lignified valve layer (endocarp b) are required for the creation of a mechanical tension in the dry silique before the detachment of the valves from the replum, that occurs in the separation layer. In particular, it has been shown that a lack of lignified and thickened secondary cell walls in the lignification layer of an *Arabidopsis* mutant silique results in the failure of seed shattering, different from the wild type, which shows fruit dehiscence [5,8,9]. Moreover, it was shown that the lack of a functional abscission layer (i.e., separation layer), along with ectopic lignification of the layer of cells that connect the valves and the replum in an *Arabidopsis* mutant, prevents silique dehiscence, as cell separation requires a specialized cell layer that is nonlignified and can undergo autolysis [6].

**Figure 2:**
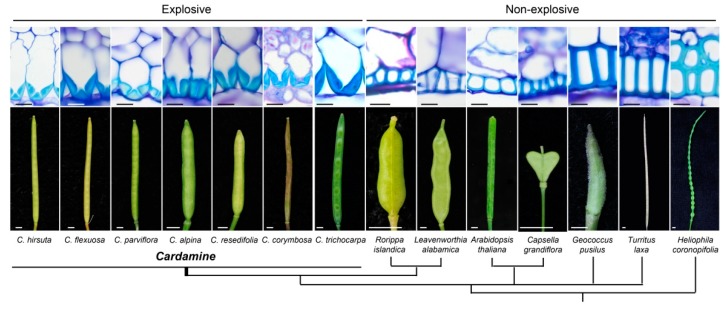
Representative patterns of secondary cell-wall lignin deposition in the endocarp b tissue for various species of the *Brassicaceae* family (as indicated) that are characterized by explosive (*Cardamine*) and non-explosive silique shattering. **Top**: Light microscopy transverse valve sections of mature fruit with cell walls stained with toluidine blue O. **Bottom**: Mature wild-type fruit. Phylogenetic relationships between species are shown in the cladogram. Scale bars: 10 mm, cells; 2 mm, fruit (reproduced with permission from Hofuis et al. [25]).

Hofhuis et al. [25] studied the lignification patterns in the silique of *Cardamine hirsuta*, a relative of *Arabidopsis* that is characterized by explosive seed shattering. They highlighted strong asymmetric lignin deposition in the endocarp b cell walls of the fruit valves as responsible for the explosive seed shattering during silique opening (Figure 2). They proposed a model in which these “hinged cells” were required to store the mechanical tension that was needed for the valve twisting. Indeed, when the dehiscence zone breaks, these hinges open, which allows the endocarp b to widen, whereby the different elasticity between the exocarp and the endocarp b is responsible for the valve curling [25].

Interestingly, Hofhuis et al. [25] compared the lignification pattern of the valves across several species of the *Brassicaceae* family, and asymmetric lignin deposition was observed only in the species of the *Cardamine* genus, which are the only ones in this family that are characterized by explosive seed shattering (Figure 2).

**Figure 3:**
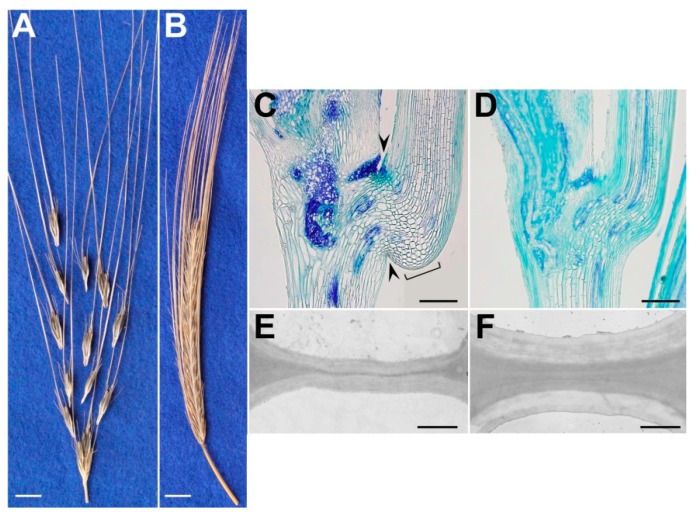
(**A**,**B**) Representative mature spikes of wild barley accession OUH602 (**A**; brittle) and induced non-brittle rachis mutant M96-1 (**B**). (**C**,**D**) Representative longitudinal sections of junction between two rachis nodes at the anthesis stage, stained with toluidine blue O. Arrowheads: separation layer (or ‘constriction groove’); square bracket: layer of expanded cells. (**E**,**F**) Representative transmission electron microscopy showing cell-wall thickness in separation layer of wild (**E**) and shattering-resistant mutant (**F**) spikes prior to disarticulation. Scale bars: 1 cm (**A**,**B**); 250 µm, (**C**,**D**); 1 µm, (**E**,**F**) (reproduced with permission from Pourkheirandish et al. [14]; with modifications).

In wild cereal species such as wheat and barley, seed shattering occurs when the spikelet detaches from the rachis, which is the central axis of the spike. This phenotype is known as brittle-rachis, as a result of which the seeds fall to the ground (Figure 3). Pourkheirandish et al. [14] demonstrated that, compared with the equivalent cell walls of the nonbrittle-rachis genotype, lower cell-wall thickness of both the primary and secondary cell walls of the separation layer (i.e., the junction where the spikelet breaks from the rachis) of wild barley results in disarticulation of the spikelets (Figure 3). This thus confirmed that conservation of both the specific tissue (i.e., the abscission layer) and the secondary cell-wall thickening is required for the modulation of shattering.

Shattering occurs in cereals also with different mechanisms, that depend on the inflorescence architecture. In rice, which produces a panicle, the grain disarticulates at the pedicel, which is the last ramification that bears the flower on the inflorescence; in this species, the correct development of a specialized abscission cell layer at the junction between the pedicel and the flower is required for grain dispersal [10,11,12,13]. Li et al. [11], reported that *Oryza nivara*, which is a wild rice species, has a continuous abscission layer between the grain and the pedicel, while the domesticated *O. sativa* had an incomplete separation layer. Moreover, they observed a stronger grain attachment to the pedicel in *O. sativa*. ssp. *japonica* accession, than in the *indica* cultivar, as, in the former, the abscission layer showed a higher degree of discontinuity [11]. It is reported that *indica* cultivars show a relatively high degree of seed shattering, while this trait was lost in several *japonica* varieties [10]. As stated by Li et al. [11], human selection favored mutations that reduced seed shattering in rice, even if the abscission layer is still partially developed also in the low shattering varieties. This process made it possible to reduce yield losses due to the seed shattering, while a certain level of grain abscission is maintained to facilitate the threshing after the harvest [11].

**Figure 4:**
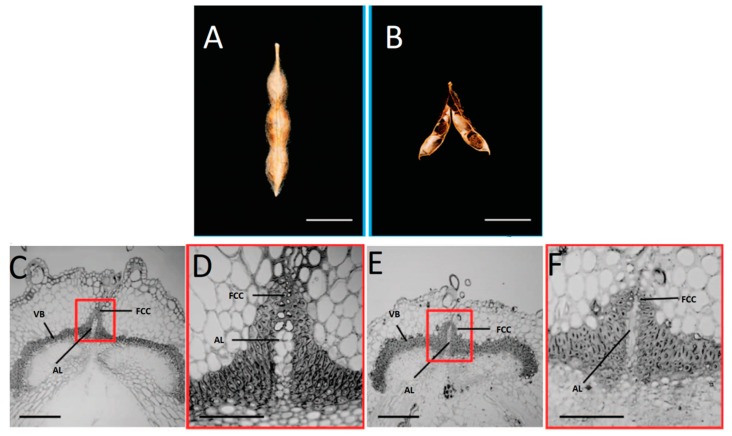
(**A**,**B**) Representative mature pods of domesticated soybean (*G. max*) (**A**) and wild soybean (*G. soja*) (**B**). (**C**–**F**) Representative cross-sections (~500 nm) of ventral sutures of domesticated (**C**,**D**) and wild (**E**,**F**) soybean pods. (**C**,**E**) Boxes: Magnified regions shown in (**D**,**F**). Details show fiber cap cells (FCC) at junctions between two vascular bundle (VB) valves, with adjoining abscission layer (AL). Scale bars: 1 cm, (**A**,**B**); 200 mm, (**C**,**E**); 80 mm, (**D**,**F**) (reproduced with permission from Dong et al. [16]; with modifications).

In legumes such as the common bean and soybean, shattering occurs when the dry fruit open along the ventral suture (Figure 4A,B). Although pods and spikes are completely different fruit, their shattering resistance appears to result from a similar and convergent mechanism. Indeed, Dong et al. [16] demonstrated that increased secondary cell-wall thickening in the fiber cap cells of the ventral suture in domesticated soybean (*Glycine max*) (Figure 4D), compared with the less-thickened cells of the wild progenitor (*Glycine soya*) (Figure 4F), leads to complete indehiscent plants, where the pods do not open along the ventral suture. Moreover, an internal lignified valve layer has been positively correlated with the shattering level in wild soybean [15], which suggested a parallelism with the lignified endocarp b of *Arabidopsis* that contributes to the modulation of shattering.

**Figure 5:**
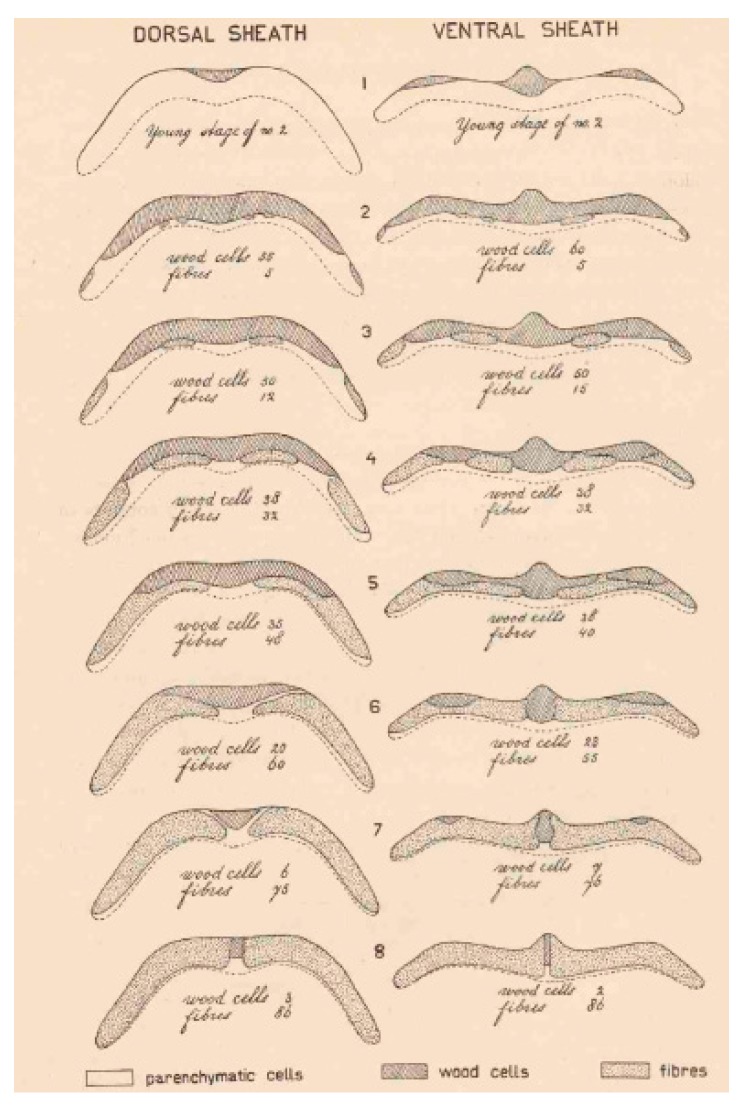
Illustration of the pod fiber content in stringless and stringy common bean varieties. Dorsal (**left**) and ventral (**right**) sheats of pods of stringy type Wagenaar (8 in the Figure), stringless type Fijne tros (2 and 3 in the Figure), intermediate F1 plants obtained after the cross between Wagenaar and Fijine tros (4, 5, and 6 in the Figure), and young pods of variety Fjine tros (1 in the Figure). The Figure shows the distribution of parenchymatic (i.e., non lignified), wood (i.e., lignified but not thickened), and fiber (i.e., lignified and heavily thickened) cells. The percentage of fiber and wood cells is shown for each pod type below the corresponding drawns (from Prakken [26]).

As has been widely reported, increased fiber content in pod sutures [20,26] and higher lignin content in pods [27] are associated with the occurrence and mode of shattering in common bean (i.e., number of twisted pods per plant). Indeed, Prakken [26] observed a high percentage of fiber cells (i.e., lignified and heavily thickened cells) in the ventral and the dorsal sheats of pods of the stringy variety Wagenaar (i.e., high shattering type), when compared with the stringless pods of the Fijne tros snap bean (i.e., indehiscent fruit), where there was a predominance of wood cells across the sheats (i.e., lignified but not thickened cells) (Figure 5).

**Figure 6:**
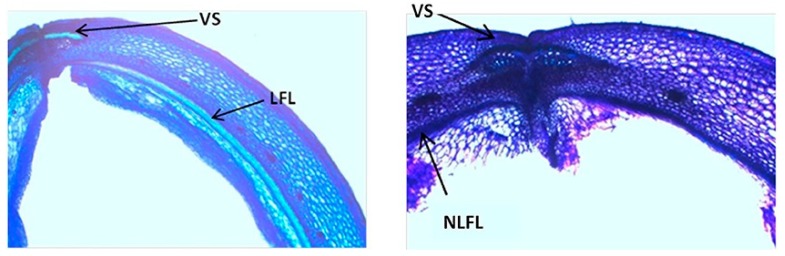
Representative light microscopy with toluidine blue O staining to illustrate differences between pod valves from the highly shattering line MG38 (**left**) and indehiscent variety Midas (**right**). Details show degree of lignification of ventral sheath (VS) and inner layer of the sclerenchymatic cells of the pod wall. LFL, lignified fiber layer; NLFL, nonlignified fiber layer. (reproduced with permission from Murgia et al. [27]; with modifications).

Murgia et al. [27], analyzed the histomorphological modifications in the pods of common bean through a comparison of highly shattering lines and the complete indehiscent variety Midas, across different developmental stages. They demonstrated that strong lignin deposition is already present at 10 days after pod setting in the cells that surround the dehiscence zone of the high-shattering RIL MG38, which is a tissue modification that can provide the mechanical tension needed to break the dehiscence zone (Figure 6, VS cells). Moreover, they observed a lignified internal cell layer in the pod valves of the high-shattering line (Figure 6, LFL cells) that is not found in indehiscent pods (Figure 6, NLFL cells). This suggested a role for this lignified tissue for both the occurrence and the mode of shattering. These findings further suggest parallelism between the lignification pattern and secondary cell-wall thickening across the dehiscent fruit of *Arabidopsis* (Figure 1), and also convergence with the valve lignification of the wild soybean [15]. On the other hand, Murgia et al. [27] showed that no clear differences can be detected in the breaking zone (i.e., the part of the pods that breaks at maturity) between shattering and non-shattering lines in common bean (Figure 6), in contrast to the previous report of Dong et al. [16] for soybean (Figure 4). However, Murgia et al. [27] did not use a specific staining for lignin, and further investigations are required to determine the phenotypic convergences at the histological level between those two related species.

Interestingly, Murgia et al. [27] also reported positive correlation between the shattering level (i.e., number of shattered pods per plant) and the valve weight, while the shattering level was negatively correlated with the 100-seed weight and with several descriptors of pod shape (i.e., pod perimeter, area, maximum width, maximum height, curved weight). They suggested an “energy cost” for the high-shattering plants due to the need for increased synthesis of molecules such as lignin and other fibers, which result in plants with heavier pods and smaller seeds. Moreover, the increased fiber content might constantly create tension during fruit development, which would lead to the formation of curved and smaller pods in the shattering lines, compared to the straighter pods of the non-shattering genotypes.

Although the same data can be explained as arising through pleiotropic effects or linkage drag, pod shattering can be viewed as a syndrome at the pod level [27].

The lignin content and tissues where there is lignification are crucial factors for shattering, along with geometrical lignin deposition in the cell walls and the environmental conditions. Indeed, some species such as *C. hirsuta* shatter because of high cell turgor of the silique [25], while other species such as legumes and *Arabidopsis* shatter after the fruit have completely dried; thus, the drier the environment, the greater their shattering susceptibility. Finally, an overview of the histological modifications that determine shattering modulation in different plant species is provided in Table 1.

## 3. Genetic Control of Seed Shattering in the Model Species *A. thaliana* and in Relevant Crops

A summary of the main genes involved in shattering in *Arabidopsis* and other relevant crop species is given in Table 2. 

**Figure 7:**
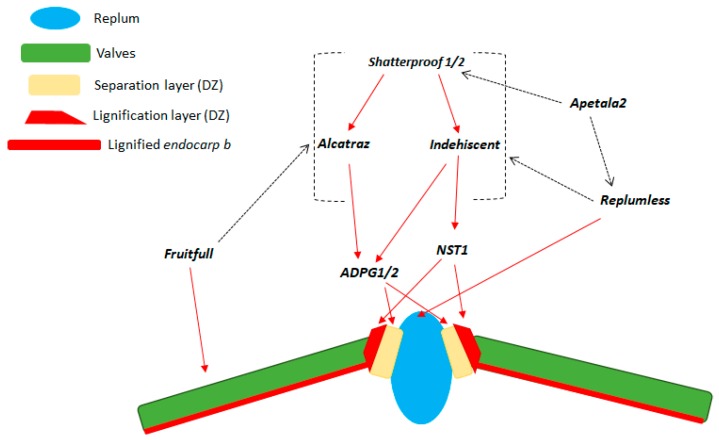
Illustration of model of the genetic cascade underlying differentiation of the dehiscence zone (DZ) and silique shattering in *A. thaliana*. Transverse section of the fruit is shown. Red arrows, positive gene regulation; black dashed arrows, negative gene regulation; square brackets, negative regulation on more than one gene (reproduced with permission from Dong and Wang [38]; with modifications).

Genetic control of shattering has been widely investigated in many crops, with several genes identified in the model species *A. thaliana*, along with their interactions in the control of the correct development of the dehiscence zone (Figure 7). Liljegren et al. [5] identified *Shatterproof-1* and *Shatterproof-2*, two MADS-box transcription factors that act at the top of a genetic cascade that directs the development of the dehiscence zone (Figure 7) [37,38].

**Figure 8:**
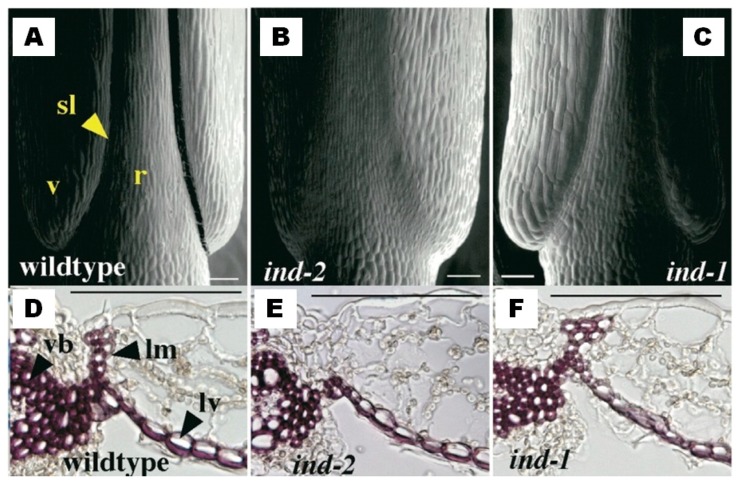
Role of Indehiscent in correct differentiation and lignification of the dehiscence zone. (**A**–**C**) Representative scanning electron microscopy of dehiscence zone in mature fruit of wild-type *A. thaliana* (**A**) and in two *A. thaliana* indehiscent mutants for *ind-2* (**B**) and *ind-1* (**C**). The *ind-2* mutant lacks correct differentiation of valve margins. sl, separation layer; r, replum; v, valves. (**D**–**F**) Representative transverse sections of the mature fruit of wild-type *A. thaliana* (**D**) and in the two *A. thaliana* indehiscent mutants for *ind-2* (**E**) and *ind-1* (**F**), with lignin-specific staining with phloroglucinol. The *ind-2* mutant lacks lignification of the valve margin cells. vb, vascular bundles; lm, lignified valve margin layer; lv, inner lignified valve cell layer. All scale bars represent 100 µm (reproduced with permission from Liljegren et al. [8]; with modifications).

The *shp1/2* double mutant showed complete indehiscent pods and a poorly-developed dehiscence zone, along with the reduced lignin content of the lignification layer [5]. *Indehiscent* [8] and *Alcatraz* [6] were identified as the target genes of *SHP*, and they promote correct differentiation of the lignification layer and the separation layer, respectively. Indeed, both the *IND* and *ALC* mutants produced indehiscent fruit, where the former (*ind-2*) completely lacks lignification in the valve margin (Figure 8). The *Fruitfull* (*FUL*) [4,39] and *Replumless* (*RPL*) [7] transcription factors confine the expression of *SHP*, *IND*, and *ALC* to the valve margins (Figure 7).

*FUL* and *RPL* are expressed in the valves and the replum, respectively, where they prevent ectopic lignification that is promoted by the valve margin genes (i.e., *SHP*, *IND*). Ripoll et al. [29] reported that replum differentiation was rescued in the triple mutant *ful rpl ap2* (*apetala2*), when compared with the double mutant *ful rpl* phenotype, which lacked correct replum development. 

Moreover, the triple mutant showed enhanced ectopic lignification in the valves when compared with the double mutant *ful rpl*, which also showed ectopic lignification in these tissues, due to the overexpression of valve margin identity genes in the valves [8,29,39]. These findings suggested that *AP2* has a role in repression of expression of both *RPL* and the valve margin identity genes (i.e., *SHP*, *IND*).

Downstream in the genetic cascade directed by *SHP*, a NAC secondary cell-wall-thickening promoting factor (*NST1*) is specifically expressed in the developing lignification layers of the dehiscence zone [9]. The *nst1* mutant completely lacks lignification of the valve margins, which results in completely indehiscent siliques [9]. Finally, the activity of two endo-polygalacturonases coded by the *ARABIDOPSIS DEHISCENCE ZONE POLYGALACTURONASE1 (ADPG1)* and *ADPG2* genes are required for the degradation of pectin in the median lamella, which promotes detachment of the valves from the replum in the separation layer prior to seed shattering [28].

Hormones have also been shown to have roles in the correct differentiation of the dehiscence zone. Interactions between *INDEHISCENT* and *SPATULA*, a basic helix-loop-helix (b-HLH) transcription factor, promote the localization of an auxin efflux carrier family protein (PIN3) in the valve margins, which leads to the minimum auxin levels needed for correct valve margin differentiation [40,41]. Marsch-Martinez et al. [42] reported that in both the *shp 1/2* and *ind* mutants, exogenous cytokinin application can partially restore the wild-type dehiscent phenotype, along with correct valve margin differentiation.

Genetic control of seed shattering has also been widely investigated in cereals. In rice, a single nucleotide polymorphism on chromosome 1 in the 5’ regulatory region of a BEL1-type homeobox gene (*qSH1*; homologous to *REPLUMLESS AtRPL* of *A. thaliana*) results in the lack of the abscission layer at the base of the rice grain, which leads to a non-shattering phenotype [10]. Zhou et al. [12] identified *SHATTERINGABORTION1* (*SHAT1*) in rice, which encodes an *APETALA2* transcription factor that is required for differentiation of the abscission zone and for seed shattering. Yoon et al. [13] identified a gene (*SH5*) homologous to *qSH1* that is positively involved in abscission zone development; its overexpression led to a reduction of the lignin deposition at the base of the spikelets, that increased shattering susceptibility. Using a fine-mapping approach, Li et al. [11] identified a mutation that is associated with a reduction of seed shattering in rice on chromosome 4, which narrowed down a previously identified QTL to a 1.7-kb region that fell within a gene (*SH4*) that is homologous to the *MYB3* transcription factor. Lin et al. [33] also identified a major dominant gene (named *SHA1*) for seed shattering on chromosome 4, which is allelic to *SH4*. In detail, a lysine residue at position 79 at both *SH4* and *SHA1* alleles determines seed shattering in rice [11,33]. Recently, Wu et al. [34] identified *ObSH4*, a gene that was under selection during the domestication of the wild African rice (*O. barthii*). A SNP mutation in the domesticated allele (*OgSH4*) of the cultivated African rice (*O. glaberrima*) resulted in the loss of the grain-shattering and led also to smaller seeds. Wu et al. [34] demonstrated that *OgSH4* in the African rice is the orthologous to *SH4*/*SHA1* in the Asian rice [11,33], suggesting the occurrence of a convergent evolution at the molecular level between *O. sativa* and *O. glaberrima*. Lv et al. [35] recently mapped a gene (*ObSH3*) in wild African rice, which encodes for a *YABBY* transcription factor. *ObSH3* is essential for the abscission zone development that is required for seed shattering, similarly to the grain disarticulation mechanism observed in the Asian rice. Lv et al. [35] demonstrated that a deletion in the genomic region that carries *ObSH3* led to the loss of seed shattering in the African rice, and they also suggested that a double mutation, i.e., the deletion of the genomic region containing *ObSH3* and the SNP mutation at *OgSH4*, evolved twice in *O. glaberrima*, in the NE (northeast inland) and the NW (northwest coastal) populations.

Lin et al. [36] identified a major gene with a complete dominance effect for seed shattering in sorghum that was named *Shattering1* (*Sh1*). *Sh1* is a transcription factor from the *YABBY* family, and the presence of three distinct haplotypes associated with the non-shattering trait in *Sorghum bicolor* suggests the occurrence of as many independent domestication events. They also demonstrated that a mutation in the orthologous in rice (*OsSh1*) determines reduction of seed shattering, that suggests a conserved function of this gene across monocotyledons. Lin et al. [36] also identified two QTLs on chromosomes 1 and 5 for seed shattering in maize, which carried *ZmSh1-1* and *ZmSh1-5*, two orthologous genes of *Sh1*, that might suggest the occurrence of a parallel evolution at the molecular level across cereals. Pourkheirandish et al. [14] identified two genes (*Btr1*, *Btr2*) in barley on chromosome 3, which are responsible for the brittle-rachis phenotype of wild barley (Figure 3) when both genes carry the dominant allele. Two independent deletion events led to neo-functionalization of both *Btr1* and *Btr2*, which resulted in barley plants with non-brittle rachis phenotype when at least one of the two genes is in the recessive form (i.e., *Btr1btr2*, *btr1Btr2*).

In soybean, which is a relative of common bean, a major gene on chromosome 16 is responsible for resistance to shattering [16]. This gene, called *SHAT1-5*, is homologous to *AtNST1*, which promotes the secondary cell-wall thickening in the dehiscence zone of *A. thaliana*. Dong et al. [16] demonstrated that the domesticated allele (*GmSHAT1-5*) is expressed in the fiber cap cells of the ventral suture at levels 15-fold those of the wild allele (*GsSHAT1-5*). The secondary cell-wall thickening that was promoted by *GmSHAT1-5* in the ventral suture of the indehiscent domesticated soybean resulted in pods that were very difficult to thresh (Figure 4), which confirmed the conserved function of *NST1* between *Arabidopsis* [9] and soybean [16] for secondary cell-wall thickening and modulation of shattering. In soybean, another gene called *PDH1* promotes the deposition of lignin in the inner sclerenchyma of the pod valves [15], which represents a histological modification that increases the shattering intensity. Here, a single nucleotide polymorphism in the domesticated allele led to a premature stop codon, which resulted in a truncated and nonfunctional protein [15].

Fourquin et al. [19] studied 17 annual species of the *Medicago* genus, eight of which were characterized by uncoiled pods, and nine of which showed coiled pods. Based on the phylogenetic relationships, all of the species that produced coiled fruit were grouped together, which suggested a common evolutionary origin within the *Medicago* genus for this trait [19]. They also demonstrated a correlation between increased lignification in the valve margins and the valve coiling predisposition, which confirmed what has been seen for other species. Furthermore, Fourquin et al. [19] identified *MtruSHP* (homologous to *AtSHP*), which was characterized by high expression in the valve margin cells. Although there was no differential expression for *MtruSHP* between the “coiling” and “not-coiling” genotypes at the beginning of valve margin lignification, this gene carries a sequence polymorphism with a signature of selection that showed complete co-segregation with the coiling/not-coiling phenotype [19].

In tomato, Vrebalov et al. [31] identified *TAGL1* (tomato *AGAMOUS-LIKE1*; homologous to *Shatterproof* (*AtSHP*)). Interestingly, down-regulation of *TAGL1* through RNA interference led to plants that produced yellow fruit due to lower carotenoid content. These fruit were also characterized by a thin pericarp and reduced ethylene synthesis, which suggested that this homolog of *AtSHP* has an important role in both fruit development and ripening in tomato [31]. *FUL1* and *FUL2* are two genes homologous to *AtFUL* that were shown to be involved in fruit ripening in tomato [43]. In particular, *FUL*1 expression was very low before the ripening stage, and then increased 10-fold after the beginning of maturation [43,44]. Furthermore, these studies also demonstrated that the *FUL1* and *FUL2* proteins can interact with the *RIN* (ripening inhibitor) protein [45], which is required for fruit ripening. Bemer et al. [44] also observed increased expression of *TAGL1* in the fruit pericarp of *FUL1/2* RNA interference lines, which suggested that the *RIN*:*FUL* protein complex has both a role in activation of the downstream ripening genes and in negative feedback on *TAGL1*. Based on this postulation, it is possible that negative regulation of *AtFUL* (homologous to *FUL1*) on *AtSHP* (homologous to *TAGL1*) has been conserved across speciation in *Arabidopsis* and tomato.

**Figure 9:**
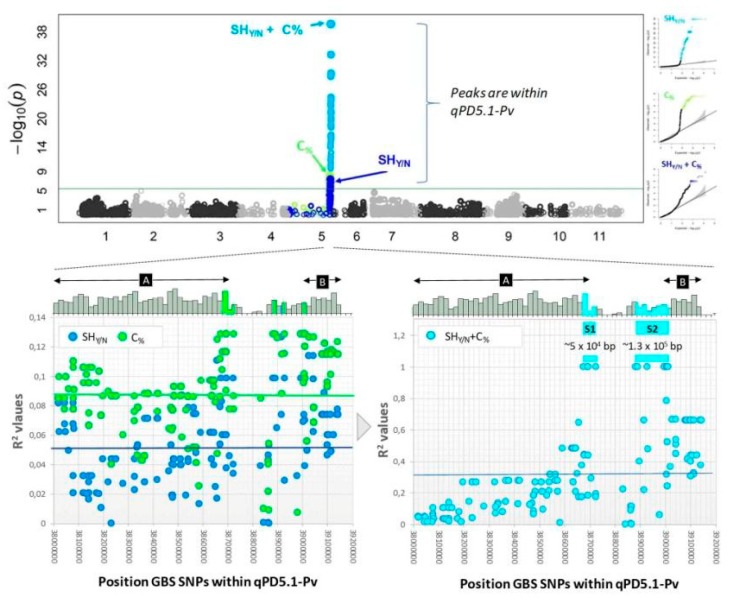
Manhattan plot from the genome-wide association study data when the trait SHyn+C% was mapped. The strongest QTL was identified for chromosome Pv05 (**top**). The single traits Shy/N and C% mapped in the same genomic regions (**bottom left**). Two sub-regions (S1, S2) co-segregated with the combined trait (SHyn+C%) with R2 = 1.00 (**bottom right**) (reproduced with permission from Rau et al. [22]).

As has been shown in soybean, *Medicago*, and tomato, a reference model can provide useful information to identify candidate genes for a trait based on the previously known gene functions. However, although genes such as *AtSHP* and *AtNST1* show conserved functions between the model species *A. thaliana* and some crops, loss of seed shattering during domestication might also have arisen after mutations at different loci. Indeed, genes homologous to *AtSHP* and *AtTIND* were identified in common bean by Nanni et al. [23] and Gioia et al. [24]. *PvSHP* [23] and *PvIND* [24] were mapped on the common bean genome to chromosomes Pv06 and Pv02, respectively; however, no co-segregation between wild and domesticated alleles for those two gene sequences and the trait shattering occurrence was observed. The gene candidate approach did not solve the question of which genes are responsible for the shattering trait in common bean. Indeed, neither *PvSHP* nor *PvIND* were clearly associated with shattering in *Phaseolus vulgaris*. Although the functions of other homologs (e.g., homologs of *AtNST1* and *AtALC* in common bean) have still not been characterized, a recent genome-wide association study suggested that mutations at nonorthologous loci formed the basis of convergent phenotypic evolution under domestication of legume species [22]. Rau et al. [22] combined pool-sequencing and genotype-by-sequencing analysis for the mapping of a population of 257 introgression lines derived through a cross between the stringless and indehiscent Andean variety Midas, and the wild Mesoamerican genotype G12873, as donor of the shattering trait. They identified a major QTL on chromosome 5 (qPD5.1-Pv) for the shattering occurrence. Also, following Murgia et al. [27], Rau et al. [22] showed that precision of the pod shattering phenotype was improved by analysis of the carbon content of the pod valves with the simultaneous consideration of two variables: the presence/absence of the trait (SHy/n), and the carbon content (C%) of the pod (Figure 9). Indeed, two genomic regions (named as S1, S2) carried 17 markers that were in a perfect association (R2 = 1.00) with the SHy/n+C% trait. qPD5.1-Pv represents a novel locus for the shattering trait in common bean, and it is positioned on a different chromosome (Pv05) to that of the *St* locus for presence of the pod string (Pv02; [20]), and to those of the *PvSHP* (Pv06; [23]) and *PvIND* (Pv02; [24]) genes.

Interestingly, in cowpea (a relative of common bean), Lo et al. [18] recently mapped two QTLs for pod shattering on chromosomes 3 and 5, named as *CPshat3* and *CPshat5*. *CPshat5* mapped in the cowpea physical map in the same position as the major locus qPD5.1-Pv in common bean, which suggested convergent evolution also at the genomic level for the loss of seed shattering between these two closely related species.

**Figure 10:**
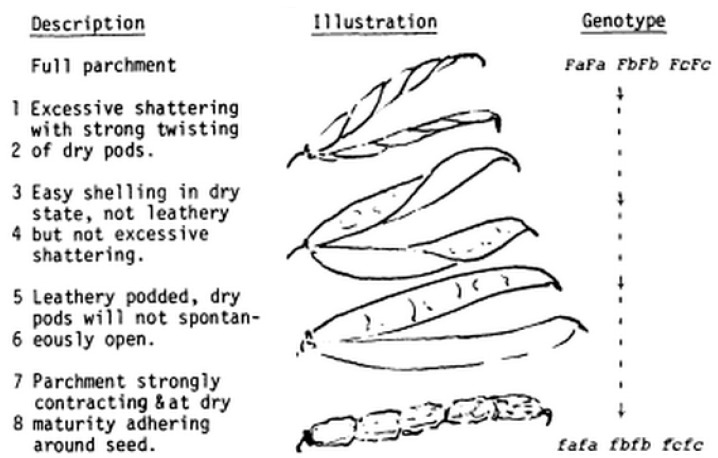
Illustration of the genetic model proposed by Lamprecht [46] for the modulation of fiber content in the pod valves and seed shattering in common bean. The model shows one major locus (FA) that controls the fiber content in the valves, with additional genes that have minor effects on the trait. Seed shattering and valve twisting are progressively reduced when recessive alleles are carried in these loci.

Moreover, in addition to the major locus on chromosome Pv05 (qPD5.1-Pv), Rau et al. [22] identified additional loci (on chromosomes Pv04, Pv05, Pv06 and Pv09) that are associated to the level (i.e., number of shattered pods per plant) and mode (number of twisting pods per plant) of shattering. They proposed a parsimonious model in which, in addition to the major locus qPD5.1-Pv, at least two other QTLs on chromosomes Pv05 (qPD5.2-Pv) and Pv04 (qPD4.1-Pv) modulate shattering intensity, which cumulatively explained 72.4% of the variability for this trait. Epistatic effects between those three QTLs were also reported, with the presence of the domesticated allele (from the variety Midas) at qPD5.1-Pv, which is associated with complete indehiscence of plants. These results are in agreement with the genetic model for the shattering trait proposed by Lamprecht [46] (Figure 10). Considering that only snap beans, which are consumed as immature pods, are fully indehiscent, the major QTL qPD5.1-Pv for seed shattering should be seen as a “late domestication” locus that was selected during the diversification of the common bean. Other loci and, in particular, the QTL on chromosome 4 (qPD4.1-Pv; Rau et al. [22]), were probably selected during the primary domestication, by the selection of allele/s for the reduction of seed shattering with hypostatic effect with qPD5.1-Pv.

## 4. Conclusions

Seed shattering has been widely investigated in the model species *A. thaliana*, and extensive information about the genetic control of this trait is available. Genes and QTLs related to seed shattering have also been identified in several crops, and the debate if the molecular pathway that underlies seed shattering is well conserved across species is advancing faster after the recent discoveries (for review, see also [47]). Although the occurrence of molecular convergent evolution leading to the same phenotypic changes for the shattering trait was proposed for rice [34,35] and across cereals [36], the same has not been well demonstrated in other crops, such as legumes. Using comparative mapping, Rau et al. [22] showed that convergent phenotypic evolution under domestication might have occurred after mutations at orthologous loci in *P. vulgaris* and *Vigna unguiculata*, which are two phylogenetically closely related crop species. 

However, this was not the case for the more distant *P. vulgaris* and *G. max*. Indeed, comparative mapping has suggested that convergent evolution of the indehiscent phenotype arose after mutations at different genes that are involved in secondary cell-wall biosynthesis and lignin deposition patterning at the pod level [22]. Moreover, Rau et al. [22] identified additional shattering related genes that were probably subject to selection during the early stages of domestication, suggesting a complex pattern of molecular variation at the basis of the genetic control of this trait in legumes. From this perspective, *P. vulgaris* and the other domesticated *Phaseolus* species represent the ideal model to study the convergent evolution during domestication because of the occurrence of at least seven independent and isolated domestication events involving five closely-related species and different gene pools of *P. vulgaris* and *Phaseolus lunatus* [48,49,50].

Wide phenotypic characterization for seed shattering has been carried out in recent years, which has confirmed the occurrence of phenotypic convergent evolution also at the histological level in different types of fruit. New efforts are required to fill the gap between the available phenotypic information and the knowledge of the genetic basis that underlies the shattering trait in the most important crops. This would ameliorate the exploitation of wild germplasm resources in plant breeding programs, and shed light on the relationships between the molecular and phenotypic variations.

## Figures and Tables

**Table 1:** Histological modifications associated with seed shattering in different plant species.

Species	Histological Modification	Observed Phenotype	Reference
***A. thaliana***	Lignification of the valve margin cells	Increase of the mechanical tension at the valve margin in the dry silique, and seed shattering	Liljegren et al. [5] Liljegren et al. [8]
***A. thaliana***	Presence of a functional abscission layer (i.e., separation layer) between valves and replum	Separation of the valves from the replum in the dry silique, and seed shattering	Rajani and Sundaresan [6]
***C. hirsuta***	Asymmetric lignin deposition in the endocarp b cell walls (internal valve layer)	Explosive seed shattering and valve curling	Hofhuis et al. [25]
***H. spontaneum*;** ***H. vulgare***	Reduced cell-wall thickness of cells of the separation layer/Cell wall thickening of cells of the separation layer	Brittle-rachis (disarticulation of the spikelet from the rachis)/Non-brittle rachis	Pourkheirandish et al. [14]
***O. nivara*;** ***O. sativa***	Presence of a continuous abscission cell layer at the junction between the pedicel and the flower/discontinuous or absent separation layer	Detachment of the grain from the pedicel and easy threshing of the panicle/reduction or loss of seed shattering	Konishi et al. [10] Li et al. [11] Zhou et al. [12] Yoon et al. [13]
***G.max***	Secondary cell-wall thickening in the fiber cap cells of the ventral suture (dehiscence zone)	Resistance to pod shattering	Dong et al. [16]
***G. soja*;** ***G. max***	Presence of an internal lignified valve layer	Increase of pod shattering	Funatsuki et al. [15]
***P. vulgaris***	Strong lignin deposition in the cells that surround the dehiscence zone	Increase of pod shattering	Murgia et al. [27]
***P. vulgaris***	Presence of an internal lignified valve layer	Increase of pod shattering	Murgia et al. [27]

**Table 2:** Genes involved in seed shattering in the model species *A. thaliana* and in relevant crops.

Species	Gene	Chromosome	Notes/Other Names	Reference
*A. thaliana*	AT3G58780	3	Shatterproof-1	Liljegren et al. [5]
*A. thaliana*	AT2G42830	2	Shatterproof-2	Liljegren et al. [5]
*A. thaliana*	AT4G00120	4	INDEHISCENT	Liljegren et al. [8]
*A. thaliana*	AT5G67110	5	ALCATRAZ	Rajani and Sundaresan [6]
*A. thaliana*	AT5G60910	5	FRUITFULL, AGL8	Gu et al. [4]
*A. thaliana*	AT5G02030	5	REPLUMLESS	Roeder et al. [7]
*A. thaliana*	AT1G32770	1	NST3, SND1	Mitsuda and Ohme-Takagi [9]
*A. thaliana*	AT2G46770	2	NST1	Mitsuda and Ohme-Takagi [9]
*A. thaliana*	AT3G57510	3	ADPG1	Ogawa et al. [28]
*A. thaliana*	AT2G41850	2	ADPG2	Ogawa et al. [28]
*A. thaliana*	AT4G36920.1	4	APETALA 2	Ripoll et al. [29]
*G. max*	Glyma.16G019400	16	Shatt 1-5	Dong et al. [16]
*G. max*	Glyma.16G141400	16	PDH1	Funatsuki et al. [15]
*S. lycopersicum*	Solyc11g010570	11	JOINTLESS	Mao et al. [30]
*S. lycopersicum*	Solyc02g071730	2	TAGL1	Vrebalov et al. [31]
*S. lycopersicum*	Solyc05g056620	5	MACROCALYX	Nakano et al. [32]
*O. sativa*	LOC_Os01g62920	1	qSH1	Konishi et al. [10]
*O. sativa*	LOC_Os05g38120	5	SH5	Yoon et al. [13]
*O. sativa*	LOC_Os04g55560	4	Shattering Abortion1, SHAT1	Zhou et al. [12]
*O. sativa*	LOC_Os04g57530	4	Shattering4, SHA1	Li et al. [11], Lin et al. [33]
*O. glaberrima*	ORGLA04G0254300	4	OgSH4	Wu et al. [34]
*O. barthii*		3	ObSH3	Lv et al. [35]
*S. bicolor*	Sobic.001G152901	1	Sh1	Lin et al. [36]
